# Regulation of Hepatocytes in G0 and G1 Phases by NOTCH3 mRNA, miR-369-3p, and rno-Rmdn2_0006 during the Initial Stage of Rat Liver Regeneration

**DOI:** 10.1155/2023/8779758

**Published:** 2023-04-27

**Authors:** Xiayan Zang, Zihui Wang, Yafei Li, Han Gao, Jianlin Guo, Wei Jin, Cuifang Chang, Juntang Lin, Kuicheng Zhu, Cunshuan Xu

**Affiliations:** ^1^College of Life Science, Henan Normal University, Xinxiang, China; ^2^State Key Laboratory Cultivation Base for Cell Differentiation Regulation, Xinxiang, China; ^3^Stem Cell and Biotherapy Technology Research Center, Xinxiang Medical University, Xinxiang, China; ^4^Academy of Medical Sciences, Zhengzhou University, Zhengzhou, China

## Abstract

The key event of liver regeneration initiation (LRI) is the switch of hepatocytes from the G0 phase to the G1 phase. This study aimed to use the data from large-scale quantitatively detecting and analyzing (LQDA) to reveal the regulation of hepatocytes in the G0 or G1 phase by competing endogenous RNAs (ceRNAs) during LRI. The hepatocytes of the rat liver right lobe were isolated 0, 6, and 24 h after partial hepatectomy. Their ceRNA expression level was measured using LQDA, and the correlation among their expression, interaction, and role was revealed by ceRNA comprehensive analysis. The expression of neurogenic loci notch homologous protein 3 (NOTCH3) mRNA was upregulated in 0 h, but the expression of miR-369-3p and rno-Rmdn2_0006 of hepatocytes did not change significantly. Meanwhile, the expression of the G0 phase-related gene *CDKN1c* was promoted by NOTCH3 upregulation, and the expression of the G1 phase-related gene *PSEN2* was inhibited by NOTCH3 downregulation. On the contrary, the expression of NOTCH3 mRNA and rno-Rmdn2_0006 was upregulated at 6 h, but the expression of miR-136-3p was downregulated. The expression of the G1 phase-related genes *CHUK, DDX24, HES1, NET1*, and *STAT3* was promoted by NOTCH3 upregulation, and the expression of the G0 phase-related gene *CDKN1a* was inhibited by NOTCH3 downregulation. These results suggested that the ceRNAs and the NOTCH3-regulated G0 phase- and G1 phase-related genes showed a correlation in expression, interaction, and role. They together regulated the hepatocytes in the G0 phase at 0 h and in the G1 phase at 6 h. These findings might help understand the mechanism by which ceRNA together regulated the hepatocytes in the G0 or G1 phase.

## 1. Introduction

The liver is an important organ of higher animals and contains hepatocytes, bile duct epithelial cells, oval cells, astrocytes, sinus endothelial cells, Kupffer cells, lacuna cells, dendritic cells, and so forth. Of them, the hepatocytes account for about 80% of the total number of liver cells and 75% of the dry weight of the liver [1]. The liver carries out various functions such as metabolism, detoxification, defense, and so forth [1, 2]. Additionally, the liver has a strong regeneration ability [3]. When the liver is injured by surgery, trauma, infection, necrosis, and so forth, the number of hepatocytes decreases sharply, and various feedback signals stimulate the hepatocytes to rapidly change from a static state to a proliferation state, so that the lost or damaged liver tissue is restored, including its structure and function, which is called as liver regeneration [4].

The noncoding RNA (ncRNA) is a class of RNA found in etonymeal organisms with important physiological functions [5]. Of these, microRNA (miRNA) is a group of single-stranded RNA with a length of about 22 nucleotides, which can bind to mRNA and inhibit its role [6–8]. For instance, Chen et al. discovered that miR-21 bound to phosphatidylinositol 3,4,5-trisphosphate 3-phosphatase and dual-specificity protein phosphatase (PTEN) mRNA, inhibited PTEN formation, and promoted hepatocyte proliferation and LR [8]. Tao et al. reported that miR-612 inhibited the proliferation and migration of hepatocellular carcinoma by acting on AKT2 mRNA [9]. Chen et al. found that miR-1 promoted cell proliferation by targeting histone deacetylase 4, but miR-133 enhanced cell proliferation by inhibiting the serum reactive factor [10].

Meanwhile, another class of ncRNA was named as circular RNA (circRNA). They are formed by the reverse splicing of the 5′ and 3′ ends of linear RNA and contain multiple miRNA binding sites, which can serve as miRNA sponges [11]. Furthermore, circRNA exhibits important physiological functions [12]. For instance, Guo et al. found that circ_03848, circ_08236, circ_13398, and circ_15013 regulated cell proliferation through binding with several miRNAs [13]. Li et al. reported that circ137 and circ2270 regulated hepatocyte proliferation by interacting with miR-127 [14]. Shang et al. found that hsa_circ_0005075 and hsa-miR-23b-5p regulated proliferation, invasion, and metastasis of the liver cancer cells through the circRNA-miRNA-mRNA axis [15].

Neurogenic loci notch homologous protein 3 (NOTCH3), a transcript factor with an N-terminal extracellular domain, an intermediate transmembrane domain, and a C-terminal intracellular domain [16–18], is one of the NOTCH family. One of its functions is to regulate cell proliferation [19–24]. For example, Tang et al. found that the inhibition of *NOTCH3* reduced the rate of cell proliferation in osteosarcoma cells [19]. Jing et al. found that increased *NOTCH3* expression led to goblet cell proliferation, while decreased *NOTCH3* expression decreased the proliferation of goblet cells [20]. Su et al. found that *NOTCH3* methylation reduced cell viability and tumor cell proliferation [21]. Hassan et al. found that nonsmall-cell lung carcinoma cells displayed high *NOTCH3* levels and cell cycle arrest when *NOTCH3* expression was reduced [22, 23]. Serafin et al. found that *NOTCH3* overexpression promoted the proliferation of colorectal cancer cells, but the cell proliferation was blocked when *NOTCH3* was inhibited [24].

This study detected the expression changes of competing endogenous RNAs (ceRNAs) by high-throughput biotechnology, analyzed their expression correlation by bioinformatics and systemic biological methods, constructed their interaction networks using Cytoscape 3.2 software, and revealed their role following the aforementioned detection and analysis to investigate their regulatory role to LR. It was found that rno-Rmdn2_0006 and miR-369-3 influenced the expression of the G0 phase- and G1 phase-related genes, which were regulated by NOTCH3 and the status of hepatocytes in the G0 and G1 phases.

## 2. Materials and Methods

### 2.1. Preparation of the Rat Liver Regeneration Model Induced by Two-Third Hepatectomy

The two-third hepatectomy (partial hepatectomy, PH) was performed as described by Higgins [25]. The male Sprague–Dawley rats (aged 10 weeks and weighing 250 ± 10 g) were used in the experiments. The liver right lobes of six rats were taken 0, 6, and 24 h after PH and mixed at the corresponding time points. At the same time, the sham operation (SO) was set up as the control. All experimental procedures in this study were carried out according to “The guidelines for the protection and use of experimental animals” published by the Ministry of Science and Technology of People's Republic of China.

### 2.2. Isolation and Identification of Rat Hepatocytes

According to Xu and Zhang [26], the rats after PH and SO were perfused under aseptic conditions at 9:00–11:00 in the morning. The hepatocytes were digested with collagenase, collected, placed in 60% saline, and centrifuged using Percoll (200 *g*, 5 min). The cell viability was evaluated using trypan blue staining. The cell purity was evaluated by the fluorescence immunochemistry of Cy3-labeled albumin (ALB) and G6P. The G0-phase hepatocytes were identified by the fluorescence immunochemistry of fluorescein (FITC)-labeled proliferating cell nuclear antigen (PCNA) and Cy3-labeled G6P. The G1-phase hepatocytes were identified by the fluorescence immunochemistry of FITC-labeled cyclin D (CCND1) and Cy3-labeled G6P. S-phase hepatocytes were identified by fluorescent immunochemistry with CCNA2 and Cy3-labeled G6P.

### 2.3. Large-Scale Quantitative Detection of mRNA

Total RNA was extracted, the ribosomal RNA was removed, the integrity of RNA was evaluated, and the mRNAs were detected. The rat genome 2302.0 chip was used for detection, and the detection was repeated three times. The ratio of gene expression at 0 and 6 h was calculated with the mRNA signal value of regenerating liver cells 24 h after PH recovery as the control [27]. The difference in mRNA expression between the PH and SO groups was calculated using the *F* test (*P* value). The genes with *P* value ≤0.05 were regarded as liver regeneration-related genes. The *t* test was used to calculate the difference in mRNA expression 0 and 6 h after PH (*P* value) [28]. When the *P* value was ≤0.05, the difference was significant; when the *P* value was ≤0.01, the difference was extremely significant.

### 2.4. Large-Scale Quantitative Detection of miRNA

The total RNA was extracted, the ribosomal RNA was removed, the integrity of RNA was evaluated, the agarose electrophoresis of samples was performed, the 25-bp RNA was recovered, the library was built, and the single-end sequencing was performed according to TruSeq Stranded Total RNA with Ribo-Zero Gold (Illumina, CA, USA). Then, the quality control of Q20, removal of splice sequences and sequences less than 15 bp and more than 41 bp in length, filtering out of reads containing N base, database analysis, sequence annotation, and quantitative detection of miRNA were conducted. The ratio of miRNA, correlation of liver regeneration (*P* value), and expression difference (*P* value) was calculated according to “materials and methods 3.”

### 2.5. Large-Scale Quantitative Detection of circRNA

Total RNA was extracted; the ribosomal and linear RNAs were removed; the integrity of RNA was evaluated; the library was built; the circRNA was sequenced; the 150/125 bp terminal pairs (reads) were obtained; the sequence was read, matched, quantified, and annotated; the sequence reliability was verified; the chromosome was located; and the circRNA was quantified. Then, the ratio value, correlation of liver regeneration (*P* value), and expression difference (*P* value) were determined according to “materials and methods 3.”

### 2.6. Prediction of G0 Phase- and G1 Phase-Related Genes of Liver Regeneration

The National Center for Biotechnology Information (NCBI) website (https://www.ncbi.nlm.nih.gov/) and Ingenuity Pathway Analysis (IPA) software were used to predict G0 phase- and G1 phase-related genes. Then, the two sets of predicted genes were integrated to get the type and quantity list of G0- and G1-related genes. Subsequently, they were compared with the meaningful expression, differential expression, and liver regeneration-related genes (tables) of “materials and methods 3,” and the genes in both tables were obtained. They were regarded as the G0/G1 phase-related genes of liver regeneration.

### 2.7. Screening of the Transcription Factors of Liver Regeneration, Which Regulated the Cellular G0 and G1 Phases

The list of transcription factors was obtained by screening the NCBI website. Then, their mRNAs were compared with the mRNA list of “materials and methods 6” of this manuscript to obtain their significant expression; a significant difference was observed in 0 and 6 h during LRI. This study selectively analyzed the NOTCH3 mRNA level and its regulation by miRNA and circRNA.

### 2.8. Prediction of G0 Phase- and G1 Phase-Related Genes Regulated by NOTCH3

The Cistrome Data Browser website and IPA software were used to predict the G0 phase- and G1 phase-related genes of NOTCH3 regulation. Then, the genes obtained from the aforementioned two tools were integrated to obtain the list of types and numbers of genes related to the G0 and G1 phases regulated by NOTCH3. Subsequently, they were compared with the meaningful expression, differential expression, and liver regeneration-related genes (tables) of “materials and methods 3” in this manuscript, and the genes in both tables were obtained. These genes were regarded as the G0/G1 phase-related genes regulated by NOTCH3 and related to liver regeneration.

### 2.9. Prediction of miRNA Binding with NOTCH3 mRNA

The IPA software and the miRwalk website (https://mirwalk.umm.uni-heidelberg.de/) were used to predict miRNA binding with NOTCH3 mRNA. The total number miRNAs, named as “theoretical miRNAs,” was listed by integrating the aforementioned two prediction lists. Finally, the “theoretical miRNA” was compared with the detection results of “materials and methods 4” in this manuscript. The miRNA with significant or extremely significant expression difference in 0 and/or 6 h was found, which was called “detection miRNA.” The distribution of the “detection miRNA” in the NOTCH family was obtained by comparing it with miRNA binding to the mRNA of each member of the NOTCH family. The miRNA that only bound to NOTCH3 mRNA was selected as “target miRNA” for analysis.

### 2.10. Interaction Network Construction of ceRNAs

In this study, miRNA-bound circRNA was predicted using miRanda software. The destination miRNA sequence was input into the miRNA (s) box of miRanda software, followed by clicking on Species, Rat, and Go to get the matching degree and binding energy between the destination miRNA and circRNA. The miRNA/circRNA pairs with matching degree (Max Score) ≥150 and binding energy (Max Energy) ≤–30 were considered as miRNA/circrna pairs. Then, compared with the results of “materials and methods 5,” circRNA with significant or very significant expression difference after 0 and/or 6 h was found, which was called “detection circRNA.” Comparing the distribution of “detection circRNA” in “destination miRNA,” we selected the circRNA that only combined with the latter, called “destination circRNA,” for follow-up analysis.

### 2.11. Interaction Network Construction of ceRNAs

The ceRNA interaction networks were constructed with Cytoscape 3.2 software. To summarize, the matches of NOTCH3 mRNA and miRNAs and their sponge molecular circRNAs bound with it were listed, and the documents were saved. Then, we clicked File-Import-Network-File and loaded the aforementioned documents into the analysis bar. At the same time, column1 was set as the analysis condition of Source Node and column2 as Target Node, following which we clicked Apply box to obtain their interaction network diagram. Then, we clicked Style-Shape to adjust the shape of the network graph, clicked Fill Color to adjust the color of the network graph, and clicked File and Import to get the network graph of interaction.

### 2.12. Statistical Analysis

In this study, the ratio values of ceRNAs were calculated in which the gene expression signal values of the control were divided by that of the experimental group. The relative ceRNAs of liver regeneration were determined using the ratio values of ceRNAs and the *F* test of SPSS 17.0 software. The expression differences of ceRNAs 0 h and 6 h after PH were analyzed using the ratios of ceRNAs and *t* tests in SPSS 17.0 software [28].

## 3. Results

### 3.1. Rat Liver Regeneration and Hepatocytes

**Figure 1:**
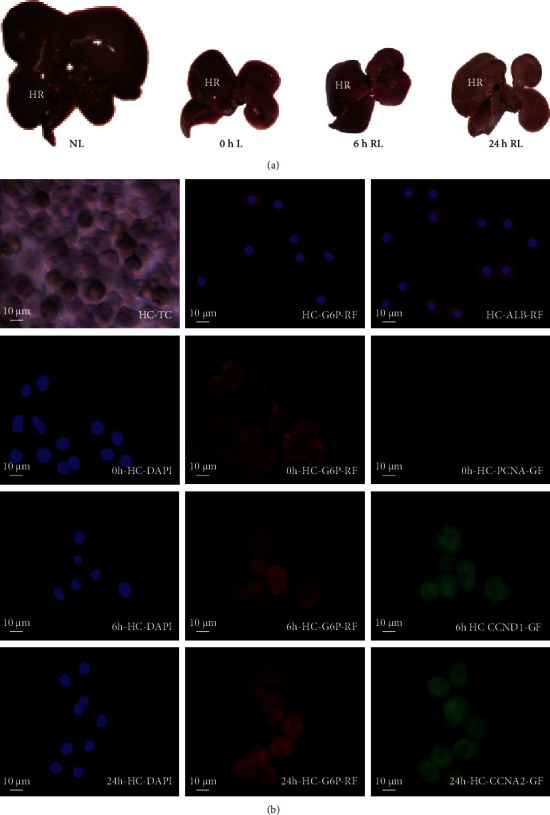
(a) Preparation of rat two-third hepatectomy (partial hepatectomy, PH) model. (b) Identification of hepatocytes. NL, normal liver of adult rats; L, liver; HR, right lobe of liver; RL, regenerated liver; HC, hepatocytes; TC, trypan blue dyeing; G6P, glucose-6-phosphatase; ALB, albumin; PCNA, proliferating cell nuclear antigen; CCND1, cyclin D1; CCNA2, cyclin A2; RF, red fluorescence; DAPI, blue fluorescence; GF, green fluorescence.

In this study, a model of rat two-third hepatectomy (PH) was prepared by the method proposed by Higgins et al. [25]. The liver right lobe of the rat was taken 0, 6, and 24 h after PH (Figure 1(a)). The hepatocytes were separated, and their mRNAs, miRNAs, and circRNAs were detected quantitatively by high-throughput biotechnology (Figure 1(b)). The results showed that the growth index of the regenerated liver was consistent with the reported findings. Also, the activity and purity of the isolated liver cells were ≥95%, and the cytochemical characteristics of the G0-, G1-, and S-phase liver cells were consistent with the report (Figure 1).

### 3.2. Interactions of NOTCH3 mRNA with miRNAs, circRNAs, and Other mRNAs of Hepatocytes

The combination of ceRNAs was analyzed using miRwalk website and miRanda software. The results showed that 131 miRNAs, which were bound with NOTCH3 mRNA, were significantly expressed 0 and at 6 h after PH. Of these, 55 miRNAs inhibited each other after 0 h and 59 miRNAs after 6 h; 33 miRNAs promoted each other after 0 h and 57 miRNAs after 6 h. On the contrary, 527 circRNAs, which were bound with miRNA, were significantly expressed 0 and 6 h after PH. Of these, 189 circRNAs inhibited each other in 0 h and 195 circRNAs after 6 h; 200 circRNAs promoted each other at 0 h and 288 circRNAs at 6 h. However, 21 genes were related to the G0 or G1 phase and were regulated by NOTCH3. Though all these genes are included in the results earlier, not all of them are discussed in detail in this article. Of these, 8 genes were inhibited by NOTCH3 at 0 h and 10 genes at 6 h, but 4 genes were promoted by NOTCH3 at 0 h and 7 genes at 6 h (Table 1).

### 3.3. Interaction of NOTCH3 mRNA with miRNAs, circRNAs, and Other mRNAs of Rat Hepatocytes

**Figure 2:**
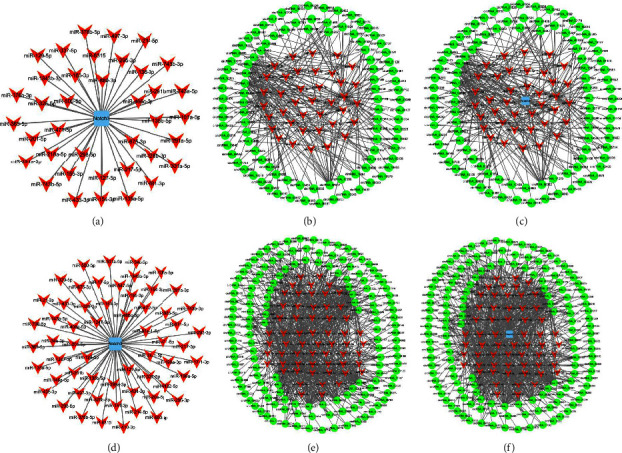
Interaction of mRNAs, miRNAs, and circRNAs of hepatocytes in G0 and G1 phases in LR. (a–c) 0 h after PH; (d–f) 6 h after PH; blue/red: mRNA-miRNA interaction; red/green: miRNA-circRNA interaction; blue/red/green: mRNA-miRNA-circRNA interaction.

The interaction of the aforementioned ceRNAs was analyzed using Cytoscape 3.2 software. The results showed that 59 miRNAs interacted with NOTCH3 mRNA at 0 h (Figure 2(a)) and 38 at 6 h (Figure 2(d)). Furthermore, 59 miRNAs and 144 circRNAs interacted to form 819 interaction pairs at 0 h (Figure 2(b)), and 38 miRNAs and 99 circRNAs interacted to form 303 interaction pairs at 6 h (Figure 2(e)). Continually, NOTCH3 mRNA, 59 miRNAs, and 144 circRNAs interacted to form 819 interaction pairs at 0 h (Figure 2(c)); however, NOTCH3 mRNA, 38 miRNAs, and 99 circRNAs interacted to form 303 interaction pairs at 6 h (Figure 2(f)).

### 3.4. Expression Correlation of NOTCH3 mRNAs, miRNAs, and circRNAs of Hepatocytes in G0 and G1 Phases

The expression correlation of miRNAs, circRNAs, and NOTCH3 mRNA of hepatocytes in the G0 and G1 phases was analyzed by system biology methods. NOTCH3 mRNA (miR-369-3p, which combined with NOTCH3 mRNA) and rno-Rmdn2_0006 (which combined with miR-369-3p) did not show significant expression change at 0 h, but rno-Rmdn2_0006 was upregulated, and miR-369-3p was downregulated at 6 h (Table 2).

The base sequence of miR-369-3p was analyzed using the miRbase software; its sequence was aauaauacaugguugaucuuu (Table 3. Meanwhile, the mother source gene of rno-Rmdn2_0006 was analyzed by its site on the chromosome and found to be *RMDN2.*

### 3.5. Expression Correlation of *NOTCH3* and NOTCH3-Regulated G0 Phase- and G1 Phase-Related Genes of Hepatocytes

The expression correlation of *NOTCH3* and the NOTCH3-regulated G0 phase- and G1 phase-related genes of hepatocytes was analyzed by the system biology methods. *NOTCH3* of hepatocytes did not show significant expression change; the expression of G0 phase-inhibited gene *CDKN1c,* which was regulated by NOTCH3, was upregulated 0 h after PH. However, the expression of *NOTCH3* of hepatocytes was upregulated; also, the expression of G1 phase-promoted genes *CHUK, DDX24, HES1, NET1*, and *STAT3,* which were regulated by NOTCH3, was upregulated 6 h after PH (Table 2).

### 3.6. Correlation with the Role of ceRNA and NOTCH3-Regulated G0 Phase- and G1 Phase-Related Genes of Hepatocytes

**Figure 3:**
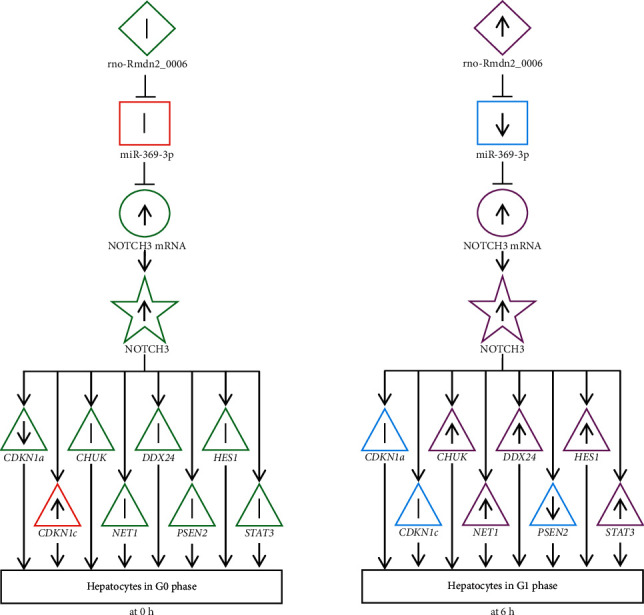
Correlation of ceRNAs and the G0 phase- and G1 phase-related genes regulated by NOTCH3 during rat liver regeneration. 0 h:0 h after PH; 6 h:6 h after PH; ◇: circRNA; □: miRNA; ○: NOTCH3 mRNA; ☆: NOTCH3; △: genes regulated by NOTCH3; ↑: gene expression upregulated; ↓: gene expression downregulated; ∣: gene expression change not significant; ⊥:inhibition; red edge: promoting G0 stage; green edge: inhibiting G0 stage; purple edge: promoting G1 phase; blue edge: inhibiting G1 phase.

This study showed that the expression of NOTCH3 mRNA, miR-369-3p, and rno-Rmdn2_0006 did not change 0 h after PH, but the expression of G0 phase-related gene *CDKN1a* was downregulated, which was inhibited by NOTCH3. Also, the expression of G1 phase-related genes *CHUK*, *DDX24*, *HES1*, *NET1*, and *STAT3* promoted by NOTCH3 was upregulated, suggesting that all of them matched with the physiological status of hepatocytes in the G0 phase (in 0 h, Figure 3). On the contrary, the expression of miR-369-3p, which inhibited NOTCH3 mRNA, was downregulated 6 h after PH. However, the expression of NOTCH3 mRNA and rno-Rmdn2_0006 was upregulated. Also, the expression of the G0 phase-related gene *CDKN1c* was upregulated, which was promoted by NOTCH3, while the expression of G1 phase-related gene *PSEN2* inhibited by NOTCH3 was downregulated, suggesting that all of them matched with the physiological status of hepatocytes in the G1 phase (in 6 h, Figure 3).

## 4. Discussion

A large number of genes, RNAs, and proteins exist in cells. Also, most physiological activities, including liver regeneration, are controlled by the content changes of the aforementioned elements and their complex interaction and regulation. Although the data obtained using biological high-throughput technology help understand the mechanism of a biological process, the related technology and methods still have limitations. In this study, the aforementioned data were used to reveal the mechanism regulating hepatocytes in the G0 or G1 phase during the rat LRI, providing inspiring results.

The hepatocytes are the main cells of liver structure and function, accounting for about 80% of the total number of hepatic cells and 75% of the total weight of the liver; they can reflect and represent most functions of the liver tissue [1]. In general, most hepatocytes of adult rat liver are in rest, named as the G0 phase. After PH was performed in rats, the hepatocytes of the remnant liver were rapidly activated and switched to the G1 phase synchronously 6 h after PH; 24 h after PH, DNA synthesis in hepatocytes continued synchronously, which was named as the S phase [29]. In this study, the two-third hepatectomy (PH), hepatocyte isolation, and biological high-throughput detection of ceRNAs were performed in a sterile room to ensure experimental reliability and avoid contamination interference. The blood in the liver tissue was removed by perfusing with PBS buffer to eliminate the blood interference in the experimental results. The ceRNA expression in hepatocytes in the S phase (24 h after PH) was used as the control of the G0 phase (in 0 h) and G1 phase (in 6 h) to analyze whether the ceRNA expression changes were meaningful in rat liver regeneration. The SO was used as the control to PH to exclude the operation influence on liver regeneration [26, 30].

Alquda et al. found that highly expressed NOTCH3 promoted cell proliferation through cell cycle proteins [31]. Kurakazu et al. found that the downregulation of *CDKN1a* led to cell cycle arrest [32, 33]. Mademtzoglou et al. found that *CDKN1c* could be used as a cell cycle repressor. The cell proliferation stopped when *CDKN1c* was highly expressed, but it was enhanced when *CDKN1c* expression was low [34, 35]. Liu et al. found that *CHUK* could promote the G1 phase of the cells [36]. Shi et al. found that *DDX24* was overexpressed in proliferating cells and underexpressed in arrested cells [37]. Rani et al. found that *HES1* induced cell proliferation. As an important member of the NOTCH signaling pathway, its reduced expression can lead to a decrease in liver regeneration levels [38–40]. Ahmad et al. found that the overexpression of *NET* led to cell proliferation, but underexpression inhibited cell proliferation [41, 42]. Bai et al. found that the overexpression of *STAT3* promoted cell proliferation, but the proliferation was arrested when *STAT3* was inhibited [43, 44]. Janicki et al. found that the overexpression of *PSEN2* led to cell arrest, but underexpression promoted cell proliferation [45–47]. The aforementioned results showed that the *NOTCH3* expression and NOTCH3 content influenced the cell phase.

Li et al. found that the low expression of miR-369-3p reduced the proliferation of chondrocytes, but the high expression of miR-369-3p promoted the proliferation of chondrocytes [48]. Bing et al. found that miR-369-3p acted on *CXCR4*, and its downregulated expression increased the number of migratory bone marrow mesenchymal stem cells [49]. Meanwhile, the prediction using IPA software, miRanda software, and miRwalk website showed that miR-369-3p bound with rno-Rmdn2_0006. However, the role of rno-Rmdn2_0006 was not reported. We noted that *RMDN2*, as the parent gene of rno-Rmdn2_0006, could regulate microtubule dynamics proteins, and research has shown that *RMDN2* is associated with the pathogenesis of lung cancer [50]. Based on this, it can be inferred that rno-Rmdn2_0006 may be closely related to cell proliferation.

The aforementioned results suggested that NOTCH3 mRNA was released 6 h after PH, which was inhibited by miR-369-3p. Furthermore, miR-369-3p could be used as an mRNA sponge to inhibit NOTCH3 mRNA translation. Hence, decreased expression of miR-369-3p resulted in more NOTCH3 protein. The latter promoted the expression of G1 phase-related genes and inhibited the expression of G0 phase-related genes; hence, the hepatocytes remained in the G1 phase. On the contrary, the NOTCH3 mRNA combined with miR-369-3p 0 h after PH. Consequently, NOTCH3 was not formed, and the promotion of the expression of G1 phase-related genes and the inhibition of the expression of G0 phase-related genes by NOTCH3 became difficult. As a result, the hepatocytes remained in the G0 phase. The results might help understand the mechanism of circRNA, miRNA, and mRNA regulating the G0 or G1 phase of liver cells.

## Figures and Tables

**Table 1:** Combination of NOTCH3 mRNA with miRNAs, circRNAs, and other mRNAs of hepatocytes.

Time point (h)	Correlation	NOTCH3 mRNA	miRNAs	circRNAs	Other mRNAs^*∗*^
0	Promotion	1	33	200	4
	Inhibition	0	55	189	8

6	Promotion	1	57	288	7
	Inhibition	0	59	195	10

	Total	1	131	527	21

^*∗*^refers to the mRNAs of genes regulated by NOTCH3.

**Table 2:** Expression correlation of *NOTCH3* and the NOTCH3-regulated G0 phase- and G1 phase-related genes of hepatocytes.

mRNA	Expression of *NOTCH3* and the NOTCH3-regulated genes			
	Control	0 h	6 h	Significance
NOTCH3 mRNA	1 ± 0	1.53 ± 1.14	2.53 ± 2.60	0.00^*∗∗*^
G0 phase-related genes				
NOTCH3-promoted				
*CDKN1c*	1.00	3.05 ± 1.27	1.08 ± 0.47	0.00^*∗∗*^
NOTCH3-inhibited				
*CDKN1a*	1.00	0.39 ± 0.07	0.93 ± 0.15	0.00^*∗∗*^
G1 phase-related genes				
NOTCH3-promoted				
*CHUK*	1.00	1.00 ± 0.17	1.52 ± 0.14	0.00^*∗∗*^
*DDX24*	1.00	0.78 ± 0.11	1.63 ± 0.11	0.00^*∗∗*^
*HES1*	1.00	1.07 ± 0.11	1.80 ± 0.16	0.00^*∗∗*^
*NET1*	1.00	0.87 ± 0.14	2.03 ± 0.06	0.00^*∗∗*^
*STAT3*	1.00	0.74 ± 0.15	2.88 ± 0.24	0.00^*∗∗*^
NOTCH3-inhibited				
*PSEN2*	1.00	1.07 ± 0.21	0.45 ± 0.06	0.00^*∗∗*^

^*∗∗*^Extremely significant.

**Table 3:** Expression correlation of ceRNAs of hepatocytes 0 and 6 h after PH.

ceRNA	ceRNA of hepatocytes			
	Control	0 h	6 h	Significance
NOTCH3 mRNA	1 ± 0	1.53 ± 1.14	2.53 ± 2.60	0.00^*∗∗*^
miR-369-3p	1 ± 0	0.70 ± 0.22	0.28 ± 0.03	0.03^*∗*^
rno-Rmdn2_0006	1 ± 0	1.26 ± 0.34	2.62 ± 0.70	0.01^*∗∗*^

^*∗*^Significant; ^*∗∗*^extremely significant.

## Data Availability

The data used to support the findings of this study are available from the corresponding author upon request.
